# Structure, Antigenic Properties, and Highly Efficient Assembly of PCV4 Capsid Protein

**DOI:** 10.3389/fvets.2021.695466

**Published:** 2021-08-24

**Authors:** Dongliang Wang, Jinhui Mai, Bo Lei, Yingjie Zhang, Yi Yang, Naidong Wang

**Affiliations:** Hunan Provincial Key Laboratory of Protein Engineering in Animal Vaccines, Laboratory of Functional Proteomics, Research Center of Reverse Vaccinology, College of Veterinary Medicine, Hunan Agricultural University, Changsha, China

**Keywords:** PCV4, Cap, VLPs, vaccine, serological diagnosis

## Abstract

Porcine circovirus type 4 (PCV4), a recently reported circovirus, was first identified in pigs with clinical signs similar to porcine dermatitis nephropathy syndrome (PDNS), in Hunan province, China, in 2019. More knowledge regarding the assembly of capsid protein (Cap) into virus-like particles (VLPs), their structure and antigenic properties, are needed to provide new knowledge for diagnosis and further characterization of PCV4. In this study, high-level expression of PCV4 Cap was achieved in *Escherichia coli* with purified Cap self-assembling into VLPs (~20 nm) *in vitro*. Furthermore, these VLPs were internalized *in vitro* by PK15 and 3D4/21 cell lines. Significant structural differences between PCV4 and PCV2 capsids were demonstrated among loops (loop BC, CD, DE, EF, and GH), based on comparisons of 3D structures. In addition, five potential B cell epitopes identified *in silico* were mostly located in surface-exposed loops of PCV4 capsid. Cross-reaction between PCV4 and PCV2 or PCV3 conferred by humoral immune responses was deemed unlikely on the basis of ELISA and Western blotting for assessment of VLPs and using PCV4 or PCV2 VLPs. In conclusion, these studies provided new knowledge regarding PCV4 capsid surface patterns. It is noteworthy that the PCV4 VLPs prepared in our study have much potential for development of serological diagnostics for PCV4 and to further characterize this virus.

## Introduction

Porcine circoviruses (PCVs) are small, spherical, non-enveloped viruses composed of circular, single-stranded genomic DNA within an icosahedral capsid. These viruses belong to the genus *Circovirus* in the family Circoviridae. PCVs have high genetic variability, with four known genotypes: PCV1, PCV2, PCV3, and a novel PCV4 genotype. Although PCV1 is considered non-pathogenic, PCV2 is the causal agent of PCV2-associated diseases (PCVADs). PCV3, first identified in 2016 by metagenomic sequencing (1), is associated with porcine dermatitis nephropathy syndrome (PDNS), cardiac disease, reproductive failure, respiratory disease, etc. (1–3).

In 2019, a newly emerging PCV4 genotype was first identified in serum samples from pigs with respiratory and enteric signs as well as PDNS, in Hunan, China (4). PCV4 is suspected to be associated with these clinical signs, which also occur in pigs infected with PCV2 or PCV3 (1). Subsequently, PCV4 infections causing clinical signs in swine were reported in Henan, Shanxi, Jiangsu, Anhui, and Guangxi provinces, China (5–8), with PCV4-positive rates ranging from 5.1% (13/257) to 25.4% (16/63). Recently, PCV4 has also been detected on swine farms in Korea (9). As distribution, epidemiology, and pathogenicity (or putative disease association) of PCV4 have not been elucidated, there is an urgent need to characterize this virus.

The genome of PCV4 is ~1.7 kb long and contains two major open reading frames (ORFs). Whereas, ORF1 encodes replication protein (Rep) involved in virus replication, ORF2 encodes the capsid protein (Cap), the sole structural protein of this virus, capable of inducing neutralizing antibodies and representing a major target for vaccine design and serological diagnosis. In the absence of an efficient method of culturing PCV4, virus-like particles (VLPs) derived from genetic and protein engineering are considered a powerful model to investigate capsid assembly, tissue tropism, and pathogenesis and, in particular, develop effective serological diagnostics for PCV4 (to clarify its incidence). In this study, structure and antigenic properties of PCV4 Cap were analyzed, and expressed PCV4 Cap protein self-assembled into VLPs in an *Escherichia coli* expression system *in vitro*. These results should promote development of effective serological diagnostics for PCV4 and increase understanding of this virus.

## Methods

### PCV4 Cap Protein Expression and Purification

A full-length PCV4 *cap* gene was codon optimized and synthesized by the GenScript Company (Nanjing, China) based on the PCV4-AHG-2019 genome sequence (GenBank accession number: MK986820). This gene was cloned into *Nde*I and *BamH*I restriction sites of the pET28a expression vector and the recombinant plasmid confirmed by DNA sequencing. To express recombinant protein, this plasmid was transformed into *E. coli* BL21 (DE3) competent cells (Biomed, Beijing, China) according to the instructions of the manufacturer. Subsequently, a single colony was selected and grown in LB medium (containing 50 μg/ml kanamycin) to an OD_600_ value reaching 0.6–0.8 before a final concentration of 0.5 mM IPTG was added to induce protein expression. After being induced, cells were grown at 25°C for 10 h, harvested, and soluble protein was purified by Ni-NTA affinity chromatography, as described (10). An *E. coli* BL21(DE3) cell pellet was placed into lysis buffer (100 mM NaH_2_PO_4_·2H_2_O, 50 mM imidazole, 10 mM Tris-HCl, 300 mM NaCl, and 100 mM KCl, pH 8.5) with 0.5 % Triton X-100, and 5 mM β-mercaptoethanol. Then, cells were disrupted by sonication (35% amplitude for 20 min), after which the homogenate was cleared by centrifugation (15,000 × g for 30 min). The supernatant was loaded on a prepacked HisTrap™ HP column (GE Healthcare Life Sciences, New York, NY, USA) according to the instructions of the manufacturer. The column was washed with wash buffer (50 mM NaH_2_PO_4_·2H_2_O, 50 mM imidazole, and 300 mM NaCl, pH 8.0), and PCV4 Cap was eluted with elution buffer (50 mM NaH_2_PO_4_·2H_2_O, 200 mM imidazole, and 300 mM NaCl, pH 8.0). Purified proteins were analyzed with SDS-PAGE and Western blot using mouse anti-6 × His tag antibody (1:3,000; Solarbio, Beijing, China) or mouse anti-PCV4 Cap serum (1:1,000).

### PCV4 VLPs Assembly and Transmission Electron Microscopy

Purified PCV4 Cap was dialyzed against phosphate-buffered saline (PBS) buffer (pH 7.4) for 48 h. The PCV4 VLPs were absorbed into carbon-coated copper grids for 10 min, stained with 1% phosphotungstic acid for 5 min, and imaged using TEM (CM100, Philips Electron Optics, Switzerland).

### Preparation of Anti-PCV4 and PCV2 Cap Serum

PCV2 VLPs were prepared as described (10). Female BALB/c mice (*n* = 4/group, 6 weeks old) were used to prepare anti-PCV4 and PCV2 Cap sera. Each mouse was initially immunized with 20 μg of PCV4 or PCV2 VLPs mixed with Freund's complete adjuvant (final volume, 0.2 ml). Booster immunizations using the same amount of VLPs mixed with Freund's incomplete adjuvant were done twice at 2-week intervals (weeks 2 and 4). Blood samples were collected 6 weeks after primary immunization, and sera were collected and stored at −20°C.

### Immunofluorescence Staining of PCV4 VLPs

Two porcine cell lines (PK15 and 3D4/21) were used to detect cell entry of PCV4 VLPs. Cells cultured in 48-well plates were incubated with 2 μg of PCV4 VLPs in each well, with DMEM, in lieu of VLPs, added to cells as a control. After incubation for 1 h, cells were washed three times with PBST (containing 0.1% Tween 20). Subsequently, cell entry of PCV4 VLPs was detected with an indirect immunofluorescence assay (IFA), as described (11). Anti-PCV4 Cap mouse serum (1:1,000) was used as the primary antibody, and immunostained slides were examined with fluorescence microscopy (Olympus BX-51, Tokyo, Japan).

### Enzyme-Linked Immunosorbent Assay

Sera of mice immunized with PCV2 and PCV4 VLPs were assessed using our established indirect ELISA, as described (10). Secondary antibody was horseradish peroxidase (HRP)-labeled goat anti-mouse IgG (Thermo Fisher Scientific, Shanghai, China). Optical densities were read at 450 nm.

### Bioinformatics Analyses

All PCVs Cap sequences in the NCBI GenBank database (http://www.ncbi.nlm.nih.gov/) were retrieved. Amino acid sequences were aligned using the ESPript 3.0 online tool (http://espript.ibcp.fr/ESPript/ESPript/). The 3D structure homology modeling was reconstructed based on PCV2 Cap protein structure (Protein Data Bank (PDB) ID: 3R0R) in SWISS-MODEL (https://swissmodel.expasy.org/) and displayed with Pymol 1.8.0.3. Linear B cell epitopes of PCV4 Cap protein were predicted by BepiPred 2.0 online tool (http://www.iedb.org/) with a default threshold of 0.5. Antigenicity was evaluated with the Vaxijen v2.0 server online tool (http://www.ddg-pharmfac.net/vaxijen/VaxiJen/VaxiJen.html).

## Results

### Expression and Purification of Recombinant PCV4 Cap

**Figure 1:**
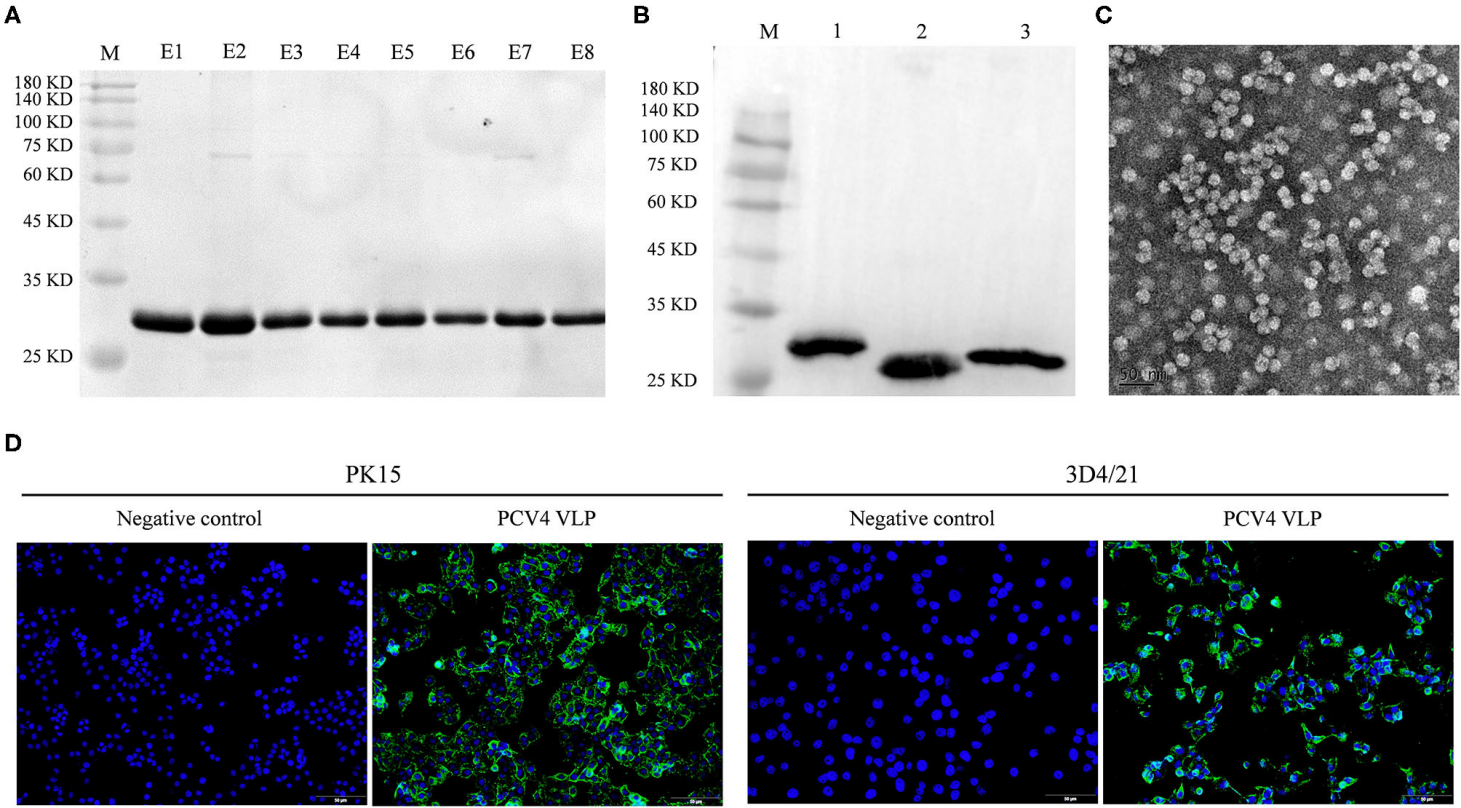
**(A)** Coomassie blue-stained SDS-PAGE of purified PCV4 Cap protein (lane M: protein marker; lanes E1–E8: purified PCV4 Cap protein). **(B)** Western blots of PCV Cap proteins using mouse anti-6 × His tag antibody (lane M: protein marker; lane 1: PCV4 Cap; lane 2: PCV3 Cap; and lane 3: PCV2 Cap). **(C)** Formation of PCV4 VLPs observed with TEM. **(D)** Entry of PCV4 VLPs into PK15 and 3D4/21 cell lines was confirmed by fluorescence microscopy. Green fluorescence represents PCV4 Cap in cells, whereas cell nuclei (blue) were stained by DAPI.

The full length of the optimized *cap* gene was cloned into a pET28a expressed vector with a 6 × His tag fused at the NH_2_ terminus of the PCV4 Cap protein to aid purification. The PCV4 Cap was successfully expressed in bacteria. Furthermore, the soluble Cap was purified with Ni-NTA affinity chromatography. The purified Cap, analyzed by SDS-PAGE, had an apparent molecular weight of ~28 kDa (Figure 1A) and was recognized by anti-His tag antibody in a Western blot (Figure 1B).

### PCV4 VLPs Self-Assembly From the Cap *in vitro*

Purified PCV4 Cap protein was dialyzed against PBS buffer (pH 7.4) to facilitate *in vitro* assembly of VLPs. Spherical morphology (diameter, ~20 nm) was observed under TEM, confirming formation of PCV4 VLPs (Figure 1C). Therefore, the Cap self-assembled into VLPs *in vitro*.

### Entry of PCV4 VLPs Into Cells

Two porcine cell lines (PK15 and 3D4/21) were used to test internalization of PCV4 VLPs into cells *in vitro*. Based on IFA, PCV4 VLPs entered both PK15 and 3D4/21 cells (Figure 1D).

### Analysis of PCV4 Capsid Structure and Predicted Immune Epitopes

**Figure 2:**
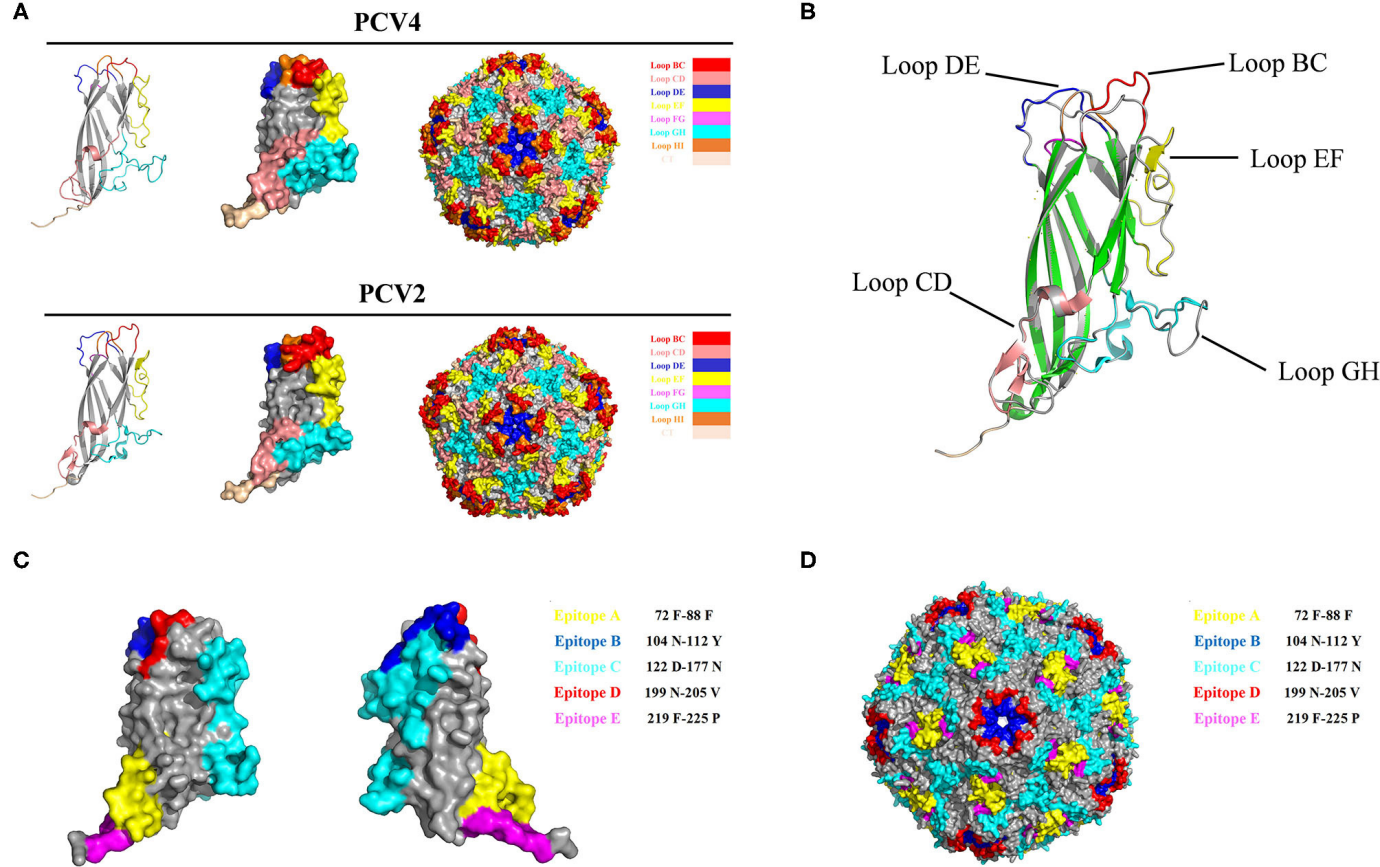
**(A)** 3D structure of PCV Caps and capsid. 3D Structure of the PCV4 Cap was simulated *via* homology modeling using the PCV2 Cap as a template (PDB ID: 3R0R). Loops were labeled with various colors. **(B)** 3D structure alignment of PCV4 and PCV2 Cap. Prediction of immune-epitopes of the PCV4 Cap **(C)** and capsid **(D)**. The five predicted epitopes are indicated by various colors. (epitope A: yellow; epitope B: blue; epitope C: cyan; epitope D: red; and epitope E: magenta).

**Figure 3:**
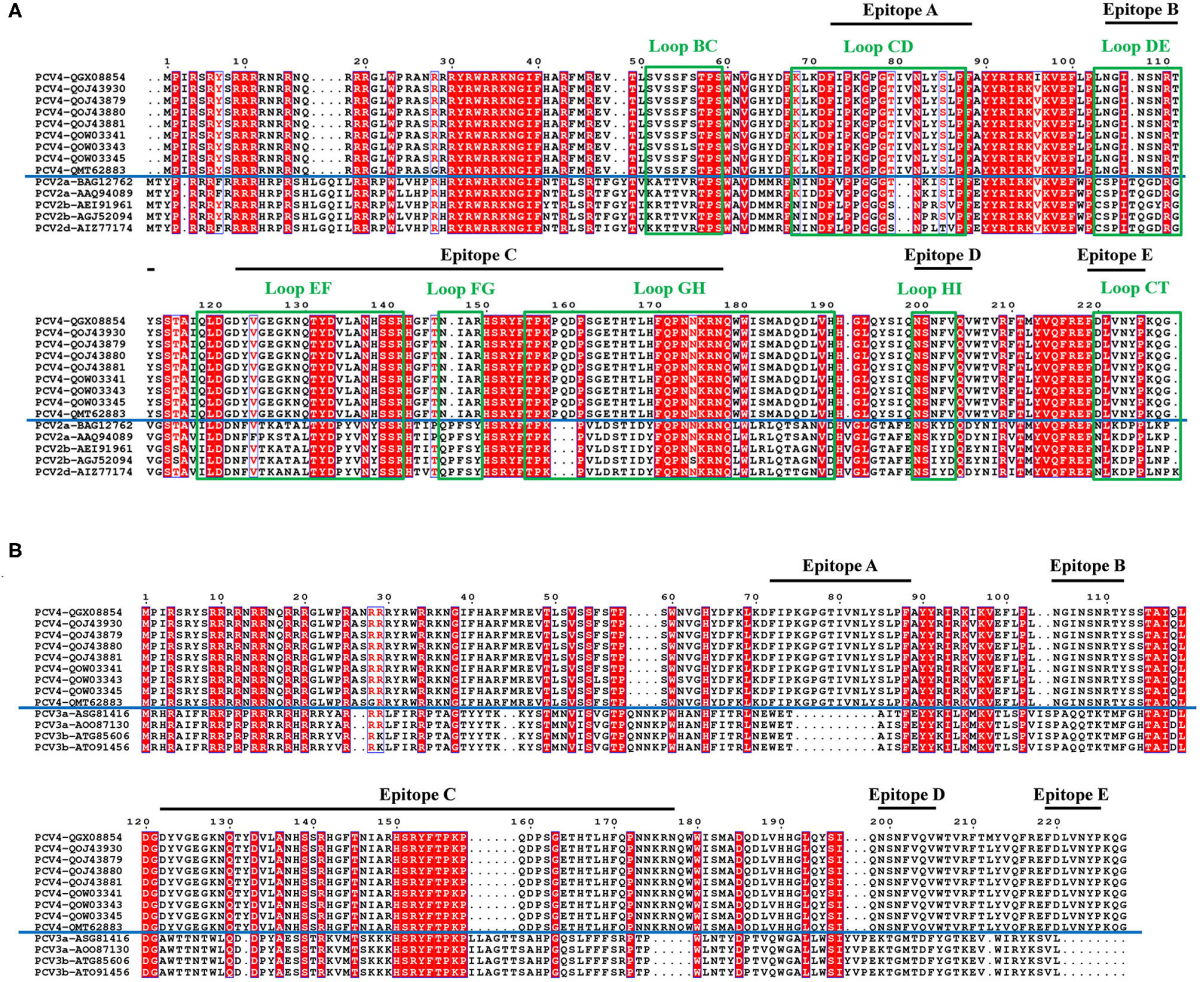
Sequence alignment of PCV Caps. Comparative amino acid sequence alignments between PCV4 and PCV2 Cap **(A)** or PCV4 and PCV3 Cap **(B)**. All loops labeled with green rectangles and black lines represent predicted PCV4 potential linear B cell epitopes.

The PCV Cap has a canonical jelly roll fold; however, Cap sequence variations among various circovirus isolates have minimal effect on the jelly roll structure. Similar to PCV2 Cap structures, a viral jelly roll structure was also observed in the PCV4 Cap (Figure 2A), based on homology modeling using a crystal structure of PCV2 Cap as a template. Based on Cap sequence alignment, most variable residues between PCV2 and PCV4 Caps were located within the surface-exposed loops, especially the icosahedral five-fold axes composed of loops BC, DE, and HI (Figures 2A, 3A). Compared to the PCV2 Cap, loops BC, CD, DE, EF, and GH were distinctly different between PCV2 and PCV4 (Figure 2B).

In the PCV4 Cap, five potential linear B cell epitopes with high antigenicity were predicted by BepiPred 2.0 based on default threshold, as indicated with various colors in the three-dimensional (3D) structure (Figure 2C). Notably, these five potential epitopes were all located in loops and mapped to the most exposed surface regions of the PCV4 capsid (Figures 2D, 3A). In-depth sequence analysis revealed the five B-cell epitopes of the PCV4 Cap shared less residue identity with either PCV2 or PCV3 Cap (Figure 3).

### Cross-Reactions Between PCV4 and PCV2 or PCV3 Cap

**Figure 4:**
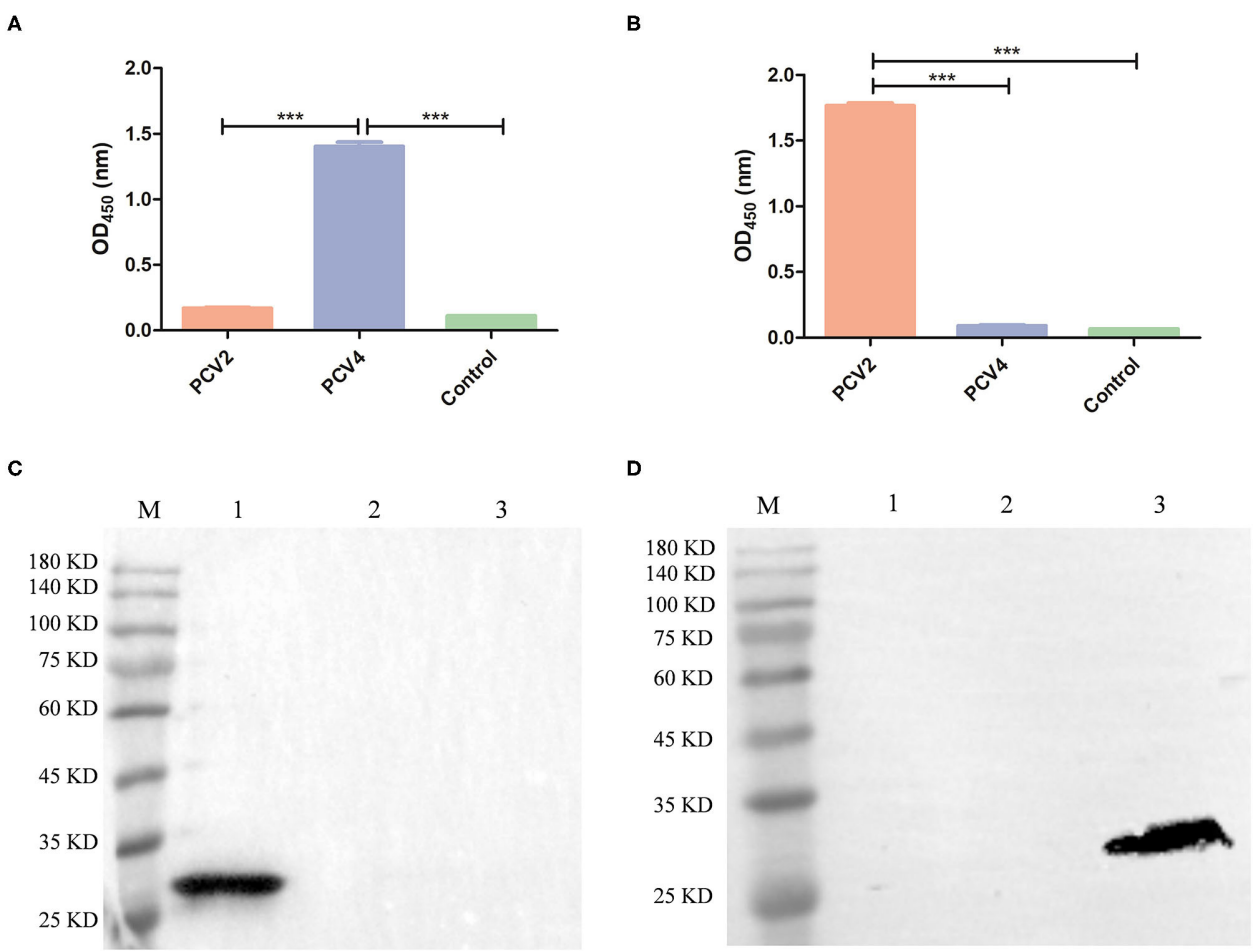
Cross-reaction between PCV4 and PCV2 by ELISA and Western blots. The reactivity of PCV4 VLP-immunized **(A)** or PCV2 VLP-immunized **(B)** mouse serum (*n* = 4) with PCV4 or PCV2 by ELISA. Reactivity of sera from PCV4 or PCV2 VLP-immunized mice, collected 6 weeks after initial immunization. Immunized mice (*n* = 2) received PBS as a control. Optical densities were read at 450 nm. ***p < 0.001. **(C,D)** Western blots of PCV Cap proteins using anti-PCV4 Cap serum or anti-PCV2 Cap serum, respectively (lane M: protein marker; lane 1: PCV4 Cap; lane 2: PCV3 Cap; and lane 3: PCV2 Cap).

PCV2 VLPs with a diameter of ~20 nm were successfully obtained (Supplementary Figure 1). Cross-reactions of anti-PCV4 or anti-PCV2 IgG from mouse serum collected 6 weeks after the primary immunization with PCV4 or PCV2 VLPs, respectively, were determined by ELISA. Sera from PCV4-immunized mice did not react with PCV2 VLP, and *vice versa*, whereas sera from PCV2-immunized mice did not react with PCV4 VLP (Figures 4A,B; Supplementary Table 1). Therefore, PCV4 immunization induced genotype-specific antibody profiles in mice. Anti-PCV4 IgG had limited cross-reaction with PCV2; furthermore, antibodies elicited by the PCV2 Cap did not react with PCV4.

Next, anti-PCV4 Cap serum was used for detecting cross-reactions with PCV2 or PCV3 Cap by Western blot, with anti-His tag antibody used as a positive control. The three PCV Caps positively reacted with anti-His tag antibody (Figure 1B). However, anti-PCV4 Cap serum recognized only the PCV4 Cap, but neither PCV2 nor PCV3 Cap (Figure 4C). In addition, anti-PCV2 Cap serum only reacted with PCV2 Cap, but neither PCV3 nor PCV4 Caps (Figure 4D). This lack of cross-reactions was attributed to the low residue identity among these Caps.

## Discussion

PCV4, a newly emerging porcine circovirus, has been identified in pigs with severe clinical disease, including PDNS-like clinical signs, diarrhea, and respiratory disease (4, 8). However, there is very limited information about its epidemiology, origin, evolution, pathogenesis, and transmission in swine herds. Notably, PCV4 has been detected as coinfections with PCV2, PCV3, or both (6–8). As PCV4 may be a serious risk for swine health, a better understanding of PCV4 pathogenesis, its epidemiology, and serological surveys to determine its prevalence is urgently needed.

VLPs are morphologically and immunogenically similar to their native viruses; furthermore, because of their ability to induce strong immune responses, they are widely used for novel vaccine design and serological diagnosis (12). Cap is the major structural protein of PCVs, and 60 Cap monomers self-assembled into VLPs *in vitro* (13, 14). The N-terminal nuclear location signal (NLS) domain of the Cap is abundant in arginine residues and contains several rare codons that limit gene expression in *E. coli*. In this study, a full-length PCV4 Cap protein via codon optimization was highly expressed in an inexpensive *E. coli* expression system. Purified Cap self-assembled into VLPs (~20 nm) *in vitro* with high yields. To characterize entry of PCV4 VLPs into cells, two porcine cell lines were used. As expected, PCV4 VLPs were capable of entering PK15 and 3D4/21 cells. Furthermore, PCV4 VLP-specific mouse serum recognized the PCV4 Cap prepared from the expression system. Therefore, PCV4 VLPs produced in *E. coli* were antigenic and well suited for future serological diagnostics and vaccine development.

We further analyzed the surface structure of PCV4 VLPs (capsid) and made comparisons to PCV2. Sequence alignment, together with 3D structure simulation, indicated substantial variation between PCV2 and PCV4 capsids in surface-exposed loop regions and surface patterns formed by various loops, although their whole structures were similar. In particular, the surface of the icosahedral five-fold axes constituted by loops BC, DE, and HI differed, as residues of these loopanalyzed the surface structures were highly divergent between PCV2 and PCV4 Caps (Figure 3A). In the PCV2 capsid, the structure of the five-fold axes was involved in conformational epitope formation. PCV2 neutralizing antibody bound to the residues around the surface of the five-fold symmetry axes, and this antibody recognized two adjacent capsid proteins as conformational epitopes (15). Diversity of the five-fold axes provided insights into antigenic differences between PCV2 and PCV4. Furthermore, these results highlighted differential structural and antigenic properties of PCV2 and PCV4 capsid proteins.

Interestingly, PCV4 Cap had high identity (70%) with the Cap of mink circovirus (4). However, it had low identities (~45 and 25%) with PCV2 and PCV3 Caps, respectively, whereas cross-reaction with PCV2 or PCV3 is unknown. To explore whether PCV4 cross-reacts with PCV2 or PCV3, anti-PCV4 Cap serum in BALB/c mice elicited by immunization with the PCV4 VLPs was used to detect cross-reaction with PCV2 or PCV3 Caps. Based on Western blot, only PCV4 Cap was recognized by anti-PCV4 Cap serum. Apparently, only PCV4 VLPs induced genotype-specific antibodies in mice. Moreover, the predicted PCV4 immune epitopes had very low identity to PCV2 or PCV3 Cap, which can further explain the absence of cross-reactions to the two Caps. Thus, cross-reaction between these Caps is unlikely, consistent with the result of the Western blot (Figure 4C). In parallel, anti-PCV2 Cap serum (produced by immunization with PCV2 VLPs) was used in Western blots to detect cross-reactions with PCV3 or PCV4 Cap; as expected, only the PCV2 Cap was specifically recognized by the anti-PCV2 Cap serum. Furthermore, using ELISA, we confirmed there was limited cross-reaction between PCV4 and PCV2. Therefore, we inferred that elicited antibody responses were unlikely to cross-protect between PCV4 and either PCV2 or PCV3.

## Conclusion

We successfully expressed the PCV4 Cap and confirmed it was capable of self-assembling into VLPs *in vitro* with a diameter of ~20 nm. To our knowledge, this is the first report of PCV4 VLPs prepared from an *E. coli* expression system. The PCV4 VLPs produced in the present study had high antigenicity, with much potential for future serological diagnostics. Importantly, cross-reactions between PCV4 and either PCV2 or PCV3 were unlikely. Finally, these PCV4 VLPs are expected to be a valuable tool to study PCV4.

## Data Availability Statement

The raw data supporting the conclusions of this article will be made available by the authors, without undue reservation.

## Ethics Statement

The animal study was reviewed and approved by the Animal Ethics Committee of Hunan Agricultural University, Hunan, China.

## Author Contributions

NW and DW conceived and designed the experiments. DW and JM performed the generation, characterization of the PCV4 VLPs, designed, and carried out the immunoassays and bioinformatics analysis. BL and YZ prepared anti-PCV4 and PCV2 Cap serum from mouse. DW, YY, and NW wrote and revised the manuscript. All authors read and approved the final manuscript.

## Conflict of Interest

The authors declare that the research was conducted in the absence of any commercial or financial relationships that could be construed as a potential conflict of interest.

## Publisher's Note

All claims expressed in this article are solely those of the authors and do not necessarily represent those of their affiliated organizations, or those of the publisher, the editors and the reviewers. Any product that may be evaluated in this article, or claim that may be made by its manufacturer, is not guaranteed or endorsed by the publisher.
