# Supplementary material for: Structure, Antigenic Properties, and Highly Efficient Assembly of PCV4 Capsid Protein
Source: Front Vet Sci. 2021 Aug 24;8:695466. doi: 10.3389/fvets.2021.695466 (PMC8421537; doi:10.3389/fvets.2021.695466)
Supplement: Supplementary file 1 [file Data_Sheet_1.docx]

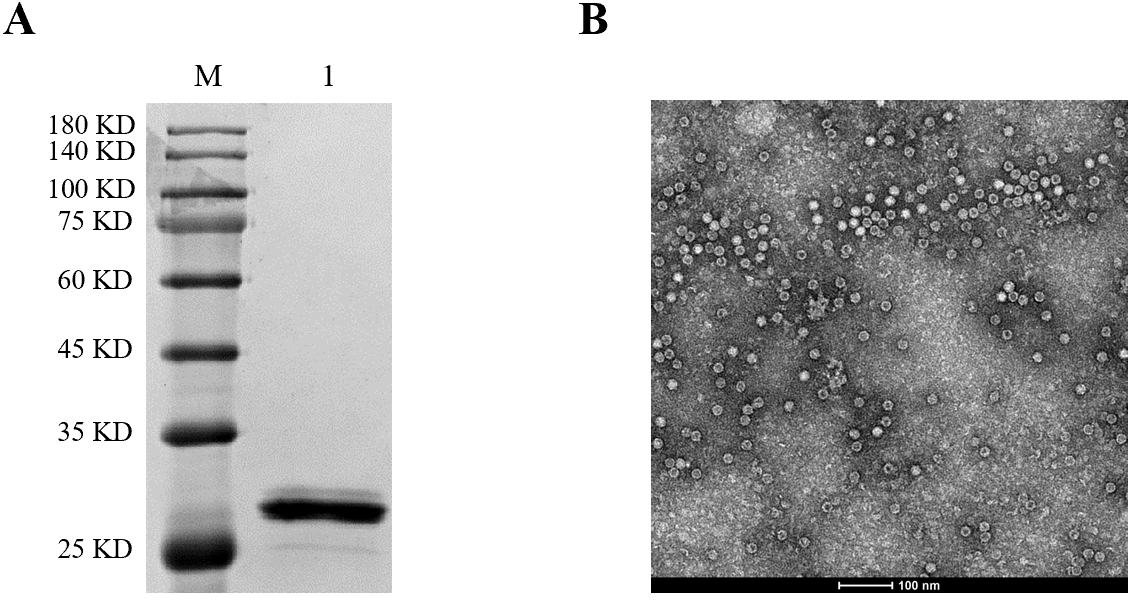


**Supplementary Figure. 1 Identification of PCV2 Cap protein and VLPs assembled *in vitro*.** (A) SDS-PAGE of purified PCV2 Cap protein (Lane M: protein marker; Lane 1: purified PCV2 Cap protein). (B) Formation of PCV2 VLPs observed with TEM.

| **Supplementary Table 1.** Reactivity of PCV4 VLP-immunized or PCV2 VLP-immunized mouse serum with PCV4 or PCV2, determined with ELISA. | | |
| --- | --- | --- |
| **Coating antigen** | **Sera** | **OD450** |
| PCV4 VLPs | PCV4 | 1.404±0.064 |
|  | PCV2 | 0.169±0.014 |
|  | PBS | 0.112±0.001 |
|  |  |  |
| PCV2 VLPs | PCV4 | 0.091±0.016 |
|  | PCV2 | 1.766±0.041 |
|  | PBS | 0.067±0.001 |
